# Improving accuracy of corneal power measurement with partial coherence interferometry after corneal refractive surgery using a multivariate polynomial approach

**DOI:** 10.1186/s12938-018-0542-0

**Published:** 2018-08-13

**Authors:** Michele Lanza, Robert Koprowski, Mario Bifani Sconocchia

**Affiliations:** 1 Multidisciplinary Department of Medical, Surgical and Dental Sciences, Campania University “Luigi Vanvitelli”, Via de Crecchio 16, 80100 Naples, Italy; 2 Department of Biomedical Computer Systems, Faculty of Computer Science and Materials Science, Institute of Computer Science, University of Silesia, Sosnowiec, Poland

**Keywords:** Refractive surgery, IOLMaster, Corneal power, Artificial intelligence

## Abstract

**Background:**

To improve accuracy of IOLMaster (Carl Zeiss, Jena, Germany) in corneal power measurement after myopic excimer corneal refractive surgery (MECRS) using multivariate polynomial analysis (MPA).

**Methods:**

One eye of each of 403 patients (mean age 31.53 ± 8.47 years) was subjected to MECRS for a myopic defect, measured as spherical equivalent, ranging from − 9.50 to − 1 D (mean − 4.55 ± 2.20 D). Each patient underwent a complete eye examination and IOLMaster scan before surgery and at 1, 3 and 6 months follow up. Axial length (AL), flatter keratometry value (K1), steeper keratometry value (K2), mean keratometry value (KM) and anterior chamber depth measured from the corneal endothelium to the anterior surface of the lens (ACD) were used in a MPA to devise a method to improve accuracy of KM measurements.

**Results:**

Using AL, K1, K2 and ACD measured after surgery in polynomial degree 2 analysis, mean error of corneal power evaluation after MECRS was + 0.16 ± 0.19 D.

**Conclusions:**

MPA was found to be an effective tool in devising a method to improve precision in corneal power evaluation in eyes previously subjected to MECRS, according to our results.

## Background

Unreliable corneal power evaluation is considered to be among the most important causes of lack of intraocular lens (IOL) power calculation accuracy after myopic excimer laser corneal refractive surgery (MECRS) [1, 2]. Currently, ophthalmologists have many different devices that are able to evaluate corneal power with high precision in eyes that did not have any surgery. However, this accuracy decreases drastically after refractive surgery, major causes of this problem are (1) the inability to calculate the exact anterior corneal curvature by current available devices because of corneal surface alteration after refractive surgery and (2) an invalidated keratometric index due to a change of relationship between the anterior and the posterior corneal surface [1, 2]. Typically, it is possible to observe an overestimation of corneal power after MECRS, which leads to an underestimation of IOL power calculated according to these data and consequently to hyperopic refraction after cataract surgery [3–5]. Hyperopia after cataract surgery is a very unsatisfactory condition. For this reason, many methods have been developed to overcome this problem [6–20]. New formulas have been introduced and new algorithms have been developed in order to provide more accuracy in IOL power calculation for these cases [21–23]. It is important to remember that the number of people who have undergone refractive surgery in the past is very high and currently the number is still increasing. It is easy to imagine that in the future most patients facing cataract surgery may have already had a previous refractive operation [24, 25]. However, a precise measurement of corneal power in patients who have previously undergone MECRS can help us to understand whether undercorrection or overcorrection is due to an error in the excimer laser calibration or in the evaluation of refraction before surgery, in order to design better ablation profiles for this type of surgery [16, 23].

One of the most widespread and reliable devices for measuring corneal power and calculating IOL power is IOLMaster (Carl Zeiss, Jena, Germany), a device that is able to measure axial length by partial coherence interferometry (PCI) and corneal power by automated keratometry, its accuracy in eyes not previously submitted to MERCS is very high but even this instrument has proved to be unreliable in corneal power evaluation after MECRS [26, 27]. By browsing biomedical engineering articles in search of methods to improve the measurements of devices, it can be observed that there is an equal preference between two approaches: those using mathematical methods for understanding and simulating phenomena in medicine and those that do not use such methods [28, 29]. The use of mathematical methods to replicate medical phenomena has certainly broadened the area of research in the field of simulation and testing. On the other hand, it is difficult to reproduce any process occurring in the living organism using the limited tools offered by mathematics [30–32]. While artificial intelligence methods still have certain limitations, they should prove useful in this field of knowledge. The purpose of this study is to improve the reliability of corneal power measurement using IOLMaster with the help of the polynomial method.

## Methods

One eye from each of 403 patients constituted the subject of the study, refractive surgery was performed in both eyes but only the right one was selected for the purpose of the study in order to avoid analysis bias due to inner correlation between pair organs. The patients, with an age range from 18 to 57 years (mean 31.53 ± 8.47 years old), were undergoing refractive surgery for myopia or myopic astigmatism. Each patient underwent a complete eye examination, corneal tomography and scans with partial coherence interferometry (IOLMaster 500, sft. ver 4.08.002, Carl Zeiss Meditech, Jena, Germany) before and 1, 3 and 6 months after photorefractive keratectomy (PRK). Demographic data of the study population with the details of the refractions and IOLMaster scans before and after PRK are summarized in Table 1. Patients with systemic and ocular diseases that might interfere with the corneal healing process or with the refractive outcome, such as diabetes, connective tissue disorders, dry eye, uveitis, corneal and lens opacities and glaucoma, were excluded from the treatment. Measurements of the sphere and cylinder was performed by combining objective and subjective refractions, thereby achieving the best-corrected visual acuity. Cycloplegic refraction was performed during the first visit, whereas subjective refraction was determined during the last visit before surgery, taking into account the cycloplegic refraction results. All surgical treatments were performed under topical anesthesia using oxybuprocaine eye drops (Benoxinato^®^ Alfa Intes, Italy) using an Allegretto Wave excimer laser system (WaveLight Laser Technologies AG, Erlangen, Germany). After surgery, the operated eye received the following medications: diclofenac sodium 0.1% eye drops twice a day for the first 2 days, moxifloxacin preservative-free eye drops until re-epithelialization and preservative-free artificial tears for 1 month. After re-epithelialization, clobetasone eye drops were prescribed for all patients for at least 1 month, four times a day. Preoperative and follow-up examinations at 1, 3 and 6 months after PRK included a comprehensive ophthalmologic examination and IOLMaster evaluation.

**Table 1:** Range, mean and standard deviation (SD) of refractions and parameters measured with IOLMaster such as axial length (AL), mean keratometry (KM), anterior chamber depth (ACD) before refractive surgery and at 6 months follow up

	Range	Mean ± SD
Age (years)	From 18 to 57	31.53 ± 8.47
Before myopic PRK		
Refraction (D)	From − 9.5 to − 1	− 4.55 ± 2.2
Axial length (mm)	From 21.86 to 30.42	25.46 ± 1.22
Mean keratometry (D)	From 37.73 to 47.85	43.88 ± 1.48
Anterior chamber depth (mm)	From 2.09 to 4.4	3.66 ± 0.31
6 months after myopic PRK		
Refraction (D)	From − 2 to + 2	+ 0.41 ± 0.5
Axial length (mm)	From 21.87 to 30.31	25.35 ± 1.19
Mean keratometry (D)	From 31.69 to 45.43	39.42 ± 2.34
Anterior chamber depth (mm)	From 2.18 to 4.29	3.56 ± 0.3

Data distributions were checked for normality by means of the Kolmogorov–Smirnov test, which showed that all data were normally distributed (p > 0.05). Statistical evaluation of the differences in the studied parameters before and after surgery was performed with SPSS (version 19.0, IBM Corporation) using the Student’s T-test for paired data. Refractions and data from IOLMaster scans performed at 6-month follow-up were used to evaluate reliability of the corneal power measurements. The data obtained in the last follow-up were selected because they should be more stable in both refraction and morphological evaluations. Parameters from IOLMaster selected for the study were axial length (AL), flatter keratometry value (K1), steeper keratometry value (K2), mean keratometry value (KM) and anterior chamber depth measured from the corneal endothelium to the anterior surface of the lens (ACD).

Effective treatment was calculated as the difference between the refractive defect that was to be completely removed and refraction measured at 6-month follow-up; these values were compared with differences in corneal power measured by IOLMaster before surgery and after 6 months. If there is a significant difference between effective treatment and corneal power changes measured by IOLMaster, it means that this device is not reliable in measuring the corneal curvature after MECRS.

This approach aims to increase precision of IOLMaster in measuring corneal power after MECRS using polynomial methods. Within the framework of the study, all combinations of these parameters were tested: AL; K1; K2; KM and ACD.

In addition, the corrected value of mean keratometry expected (KM_R_) was calculated. This value represents the one that should have been observed after MECRS in the evaluated eyes. It has been calculated adding to the mean keratometry (KM) measured before surgery to the effective treatment. The number of defined and analyzed parameter combinations was limited to five:

- AL, K1, K2, ACD;
- AL, ACD;
- AL, K1, K2;
- AL;
- K1, K2.

The selection of these combinations has been adopted taking in account parameters usually measured and utilized in formulas for IOL calculation, moreover there are the parameters more often able to provide indications about refractive defect of eyes. For these reasons, combinations including only one keratometric value, such as K1 or K2, have not been purposed: they could bias the overall analysis: these values alone could not provide reliable indications about corneal power.

A multivariate polynomial approach was used, which fits a general polynomial regression model in n dimensions. The value of n dimensions was changed in the range n∈(1,7). The proposed range results from the limitations adopted for the distribution of patients’ parameters. The approximation of the relationships between AL, K1, K2, ACD and the resulting KM_R_ is not linear [33], therefore the value of n dimensions of the polynomial regression model must be above one. On the other hand, too high *n* values will result in overfitting the data. This analysis was performed using the author’s (Koprowski) algorithm which works fully automatically and repeatedly, allowing for quantitative results. This algorithm was written in MATLAB Version 7.11.0.584 (R2010b), Operating System: Microsoft Windows 7 Version 6.1 (Build 7601: Service Pack 1), Java VM Version: Java 1.6.0_17-b04 with Sun Microsystems Inc. Java HotSpot (TM) 64-Bit Server VM mixed mode, with: Bioinformatics Toolbox, Version 3.6 (R2010b); Image Acquisition Toolbox, Version 4.0 (R2010b); Image Processing Toolbox, Version 7.1 (R2010b); Neural Network Toolbox Version 7.0, (R2010b); Statistics Toolbox, Version 7.4 (R2010b) and Wavelet Toolbox Version 4.6 (R2010b). The calculations lasted no more than one second on a Windows-based PC and the Intel i7-3770 processor.

All procedures performed in studies involving human participants were in accordance with the ethical standards of the institutional and/or national research committee and with the 1964 Helsinki declaration and its later amendments or comparable ethical standards. For this type of study formal consent is not required because patients signed an informed consent for the surgical procedures that included use of data for following eventual studies.

The datasets used and/or analysed during the current study are available from the corresponding author on reasonable request.

## Results

The results were obtained in two stages:

- Data pre-processing and,
- Multidimensional polynomial approximation.

### Data pre-processing

**Fig. 1:**
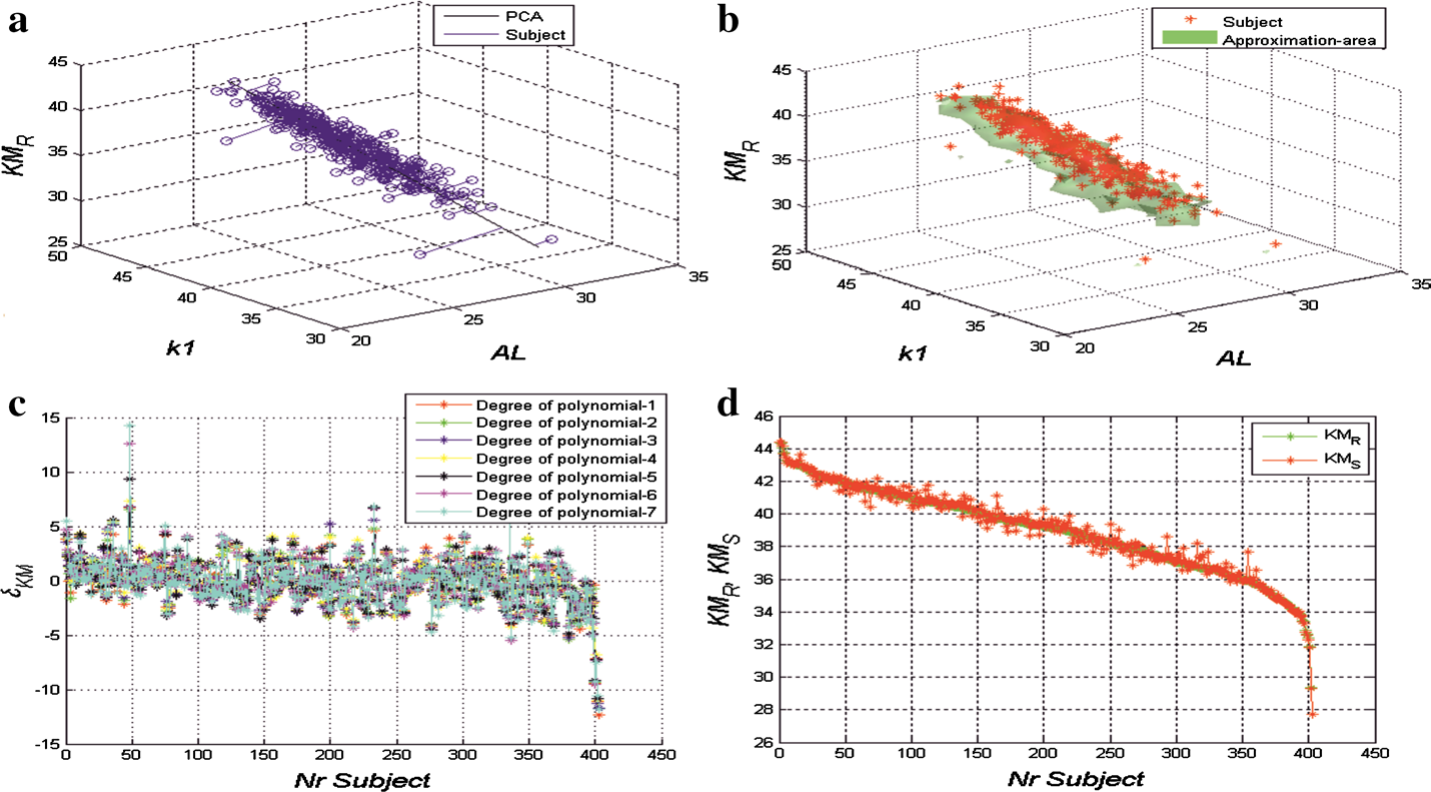
**a** Graph showing how principal component analysis (PCA) can be used to approximate the distribution of the variable mean keratometry expected (KM_R_) as a function of variables flatter keratometry (K1) and axial length (AL). Consecutive patients are marked in blue, while PCA approximation is in black. **b** Graph showing how the distance function in the three-dimensional space is used to approximate the distribution of the variable mean keratometry expected (KM_R_) as a function of variables flatter keratometry (K1) and axial length (AL). Consecutive patients are marked in red, while the approximation area is in green. **c** Graph of changes in δs_KM_ for 403 consecutive patients and 7 different polynomial degrees. It is possible to observe that the error values are quite large and vary widely. Of the seven polynomial degrees, n = 7 was chosen (for example degree of polynomial 1 is red, degree of polynomial 2 is green etc.). **d** Graph of changes in mean keratometry expected (KM_R_) and mean keratometry predicted (KM_S_) for 403 individual patients (arranged in order from the highest values of KM_R_)

In the initial stage, the changes in KM_R_ values as a function of K1 and AL were visualized. The values of K1 and AL were selected arbitrarily only for the visualization of the changes and preliminary assessment of the complexity of the problem. This visualization, shown in Fig. 1a, b, was supplemented with principal component analysis (PCA) and analysis of clusters based on the analysis of the distance between the data of individual patients. In the first case (Fig. 1b), one main component was proposed—marked with a black line in Fig. 1a. The calculated coordinates K1, AL and KM_R_ of the extreme points for all analysed patients are 29.1, 30.5 and 29.5 and 23.1, 43.8 and 44.6. The assessment of the possibility of approximating this distribution with one main component was performed by defining the error δ_KM_(i) for a single patient and the error δs_KM_(i) as the mean error for all *I* patients:

1 $$\delta_{KM} \left( i \right)\, = \,\left| {KM_{R} \left( i \right)\, - \,KM_{S} \left( i \right)} \right|$$

2 $$\delta_{sKM} \, = \,\frac{{\mathop \sum \nolimits_{i = 1}^{I} \left| {KM_{R} \left( i \right)\, - \,KM_{S} \left( i \right)} \right|}}{I}$$

where: KM_R_: true data (ground true)—the value of KM calculated for the patient after correction, calculated taking into account effective treatment after surgery, KM_S_: prediction data—the value predicted using the approach discussed, i: subject number.

The error of fitting one main component to the corrected data (KM_R_) is 2.3 + 1.1 D (mean error for all I subjects with standard deviation). For the second approach described, measurement of distances for each point (between ground true and prediction data) in the three-dimensional space was proposed. This enabled the creation of an approximated area covering a cluster of points, where the extreme values of the parameters were rejected. The resulting three-dimensional object allows for predictions of KM_R_ values based on the parameters K1 and AL (Fig. 1b). The values of the error δs_KM_ in this case exceeded 3D. Therefore, the polynomial approach was proposed for use.

### Multidimensional polynomial prediction

In the polynomial approach, the value of n dimensions was changed in the range n∈(1,7). The results of the error δs_KM_ for all 403 patients and parameters AL, K1, K2 and ACD are shown in Fig. 1c, where it is possible to observe that the error values are quite large and vary widely. The minimum value is − 13 and the maximum one is + 15 D. Of the seven polynomial degrees, n = 7 was chosen. The results obtained for all the parameters and polynomial approach are shown in Fig. 1d. The KM_S_ prediction values are very close to KM_R_ values. The quantitative results are shown in Table 2 (the training and test sets).

**Table 2:** Mean values of the error *δ*_*SKM*_ (and standard deviation) between real and predicted data *KM* for different combinations of parameters and different polynomial degrees (for 403 patients constituting the training and test sets)

Parameters: degree (*n*)	*AL, K1, K2, ACD*	*AL, ACD*	*AL, K1, K2*	*AL*	*K1, K2*
1 (D)	0.68 ± 0.66	1.0 ± 0.89	0.69 ± 0.67	1.0 ± 0.93	0.77 ± 0.72
2 (D)	0.65 ± 0.64	1.0 ± 0.85	0.66 ± 0.66	1.06 ± 0.91	0.75 ± 0.72
3 (D)	0.62 ± 0.608	0.98 ± 0.81	0.65 ± 0.63	1.0 ± 0.89	0.74 ± 0.72
4 (D)	0.59 ± 0.57	0.95 ± 0.80	0.64 ± 0.61	1.03 ± 0.89	0.73 ± 0.71
5 (D)	0.52 ± 0.505	0.94 ± 0.80	0.60 ± 0.60	1.03 ± 0.89	0.73 ± 0.70
6 (D)	0.35 ± 0.39	0.93 ± 0.79	0.55 ± 0.59	1.0 ± 0.89	0.71 ± 0.70
7 (D)	0.227 ± 0.25	0.92 ± 0.80	0.50 ± 0.56	1.0 ± 0.89	0.69 ± 0.68

*AL* axial length, *K1* flatter keratometry value, *K2* steeper keratometry value, *KM* mean keratometry value, *ACD* anterior chamber depth measured from the corneal endothelium to the anterior surface of the lens

The table presented (Table 2) shows that the smallest error values are for n = 7 and four parameters AL, K1, K2 and ACD, and are 0.227 ± 0.25 D. The largest values of δ_SKM_ are obtained for AL and n = 2, i.e. 1.06 ± 0.91. By analysing this table with the results, it can be assumed that the next polynomial degrees n = 8, n = 9 etc. will increase the predictive accuracy of KM values. Since overfitting data is very likely in this case, the set of 403 patients was divided into two subsets: the training set comprising 2/3 of the total number (269 patients) and the test set comprising the remaining one-third (134 patients). The division of the sets results from the typical proportions applied, e.g. in machine learning and classification [the objects were randomly selected—a random generator was created matrix containing pseudorandom values drawn from the standard uniform distribution on the open interval (0,1)]. The training set is the basis for calculating polynomial coefficients, whereas the values of δ_SKM_ are calculated for the test set. The results are shown in Table 3.

**Table 3:** Mean values of the error *δ*_*SKM*_ (and standard deviation) between real and predicted data *KM* for different combinations of parameters and different polynomial degrees (for the test set)

Parameters: degree (*n*)	*AL, K1, K2, ACD*	*AL, ACD*	*AL, K1, K2*	*AL*	*K1, K2*
1	0.71 ± 0.53	1.01 ± 0.84	0.71 ± 0.52	1.02 ± 0.87	0.78 ± 0.66
2	0.16 ± 0.19	0.86 ± 0.14	0.31 ± 0.24	1.06 ± 0.48	0.28 ± 0.38
3	0.73 ± 0.61	1.06 ± 0.92	0.74 ± 0.56	1.04 ± 0.93	0.78 ± 0.66
4	0.83 ± 0.33	1.26 ± 1.04	0.93 ± 0.33	1.23 ± 1.01	0.99 ± 1.25
5	0.70 ± 0.55	1.46 ± 1.42	1.04 ± 0.53	1.21 ± 1.03	1.28 ± 1.34
6	1.02 ± 0.41	1.77 ± 0.95	1.03 ± 0.46	1.43 ± 0.99	1.44 ± 1.20
7	1.23 ± 0.78	1.99 ± 1.23	1.24 ± 0.66	1.42 ± 1.43	1.36 ± 1.34

*AL* axial length, *K1* flatter keratometry value, *K2* steeper keratometry value, *KM* mean keratometry value, *ACD* anterior chamber depth measured from the corneal endothelium to the anterior surface of the lens

According to the above table (Table 3), the mean error in calculating corneal power after MECRS using AL, K1, K2, ACD and n = 2 is + 0.16 ± 0.19 D. Above the second polynomial degree, there occurs the problem of overfitting data. For this reason, for the same training and test data (as was the case with the results shown in Table 2) for n = 5, 6 and 7, the results obtained were apparently incrementally better. On the other hand, when the division into training and test data was applied, the actual error was + 0.16 ± 0.19 D, this is representing the error in corneal power evaluation after MERCS when this method was applied. When analysing Table 3, it should be noted that the highest error values were obtained for the parameters AL and ACD as well as AL. This is also confirmed by the results presented in Table 2.

Example: formula for variable K1, K2 and 1 order polynomial:

3 $$KM_{S} \, = \, - \,1.0359\, + \,K1\,*\,0.4345\, + \,K2\,*\,0.5769$$

The formula proposed by the authors to calculate KM_S_ (variable AL, K1, K2. ACD and 2 order polynomial – please see Table 3).

4 $$\begin{aligned} KM_{S} & \, = \,11.12\,*\,AL\, - \, 4.51\,*\,K1\, + \, 8.1\,*\,K2\, - \,21.09\,*\,ACD\, + \, 0.0061\,*\,AL\,*\,K1 \\ & \quad - \,0.115\,*\,AL\,*\,K2 \, + \,0.521\,*\,AL\,*\,ACD\, + \, 0.013\,*\,K1\,*\,K2 \\ & \quad - \,0.034\,*\,K1\,*\,ACD\, + \, 0.235\,*\,K2\,*\,ACD\, - \,0.183\,*\,AL^{2} \\ & \quad + \, 0.054\,*\,K1^{2} \, - \, 0.075\,*\,K2^{2} \, + \, 0.034\,*\,ACD ^{2} \, - \,143.7 \\ \end{aligned}$$

## Discussion

Cataract surgery is no longer perceived as sight-saving surgery but as a refractive operation. Patients do not only expect vision improvement following surgery but they often demand better quality of vision, free of spectacles [24]. A satisfactory refractive result, represented by a postoperative refractive defect in spherical equivalent ± 0.5 D and astigmatism < 1 D is the goal, in many cases, of phacoemulsification and IOL implant but only 55% of the eyes that undergo this surgery will achieve this result [25]. Moreover, patients who have previously undergone MECRS have high expectations of improved vision without spectacles but it is much harder in these eyes to achieve emmetropia after cataract surgery [25, 34, 35].

Patients who underwent myopic refractive surgery often have a change in the optical profile of the cornea and variable degrees of astigmatism [35]. When these eyes, whose anatomy is modified by an excimer laser, develop cataracts, there may be very disappointing hyperopic results due to difficulties in calculating the IOL power to be implanted during cataract surgery [35].

The ideal method to estimate accurate corneal power is to measure it directly with a reliable device. IOLMaster is a very widespread and easy to use device, and once its precision in corneal power evaluation after MECRS has improved, it will provide ophthalmologists with a satisfactory solution to this problem without any additional cost for offices or hospitals.

Different correcting formulas have been proposed in order to improve reliability of corneal power evaluation after MECRS [8–20] and most of them rely on linear regression calculation of the correcting factors that still produce a discrete lack of accuracy. In order to deal with the increasingly high expectations of patients, especially for those who have undergone MECRS in the past, physicians need to improve their tools to achieve emmetropia after cataract surgery. For this reason, a more sophisticated method to calculate corneal power would be a very good option in this field. The multivariate polynomial one, was selected because of its reliability. In order to obtain the best results, it is fundamental to remember that this strategy may produce a wide range of error if proper polynomial degree analysis is not chosen (Fig. 1c). In this study, 7 degrees were selected. It is important to emphasize that these calculations were performed on 403 eyes, which is a very substantial population. It also takes into account morphological and refractive data at 6 months follow up, when the effects of surgery may be considered to be stable.

This study is purposing a new approach to accurately calculate corneal power after MERCS, it has been developed thanks to data obtained before and after myopic PRK but it is working only using the IOLMaster parameters after this kind of surgery. Further studies need to verify the clinical reliability of this methods and, if the theoretical results will be confirmed in clinical practice, it will support previous researchers’ theory stating that nowadays it is no longer necessary to obtain patient data before MECRS to provide an accurate IOL power calculation: many alternative methods and formulas have been tested to be reliable [36]. The next stage of our research will be to apply this method to actual cases of patients who will refer to our practices for cataract surgery after MECRS. This will enable to compare the results obtained with the most widespread and reliable formulas and methods used in these cases.

In conclusion, this study provides a new formula for corneal power calculation after MECRS using IOLMaster. This device has been proven to be very reliable and accurate in naïve eyes but has shown a lack of precision in eyes previously subjected to MECRS. The proposed correcting formula has demonstrated very satisfactory potential precision as a result of the elaborated analysis strategy chosen to obtain it.
